# A cross-sectional study on turnover intention of nurses in eastern China

**DOI:** 10.1186/s12913-024-10849-9

**Published:** 2024-04-03

**Authors:** Haolian Huang, Liping Wang, Ruilian Qian, Yanhong Zhang

**Affiliations:** 1 Department of Nursing, Affiliated Nanjing Brain Hospital, Nanjing Medical University, Nanjing, China; 2 Department of Geriatric Neurology, Affiliated Nanjing Brain Hospital, Nanjing Medical University, Nanjing, China

**Keywords:** Nurse, Turnover intention, Influencing factors, Career development, Stratified analysis

## Abstract

**Background:**

This study aimed to investigate the turnover intention among nurses in eastern China and explore the association between turnover intention and personal characteristics, family factors, and work-related factors.

**Methods:**

A total of 2504 nurses participated in a cross-sectional survey administered in 26 hospitals in Eastern China from October to November 2017. In December 2021, a survey was conducted on nurses who resigned between December 2017 and November 2021.

**Results:**

The turnover intention score of in-service nurses was 15 (12–17), and 43% of nurses had a high turnover intention, which was mainly due to the following reasons: age < 40 years, raising two or more children, monthly income of USD786.10-1572.20 or < USD786.10, occupation was assigned or selected according to parental wishes, ≤ 1 or ≥ 2-night shifts per week, contractual or third-party personnel agents, full-time nurses with part-time jobs, and high job stress. Among 102 retired nurses, 80.4% reported family reasons for leaving, 39.2% for work reasons, and 21.6% for other personal reasons.

**Conclusion:**

Nurses’ intention to leave their occupation is high in Eastern China. Age < 40 years old, > 1 child, low income, involuntary career selection, frequent night shifts, informal employment, part-time, and high job stress are significant factors associated with nurses’ willingness to leave. Government and hospital administrators should consider ways to address these factors to retain nurses in hospitals in eastern China and improve the quality of nursing services.

**Supplementary Information:**

The online version contains supplementary material available at 10.1186/s12913-024-10849-9.

## Background

The nurse shortage is a global problem. According to the World Health Organization (WHO), the worldwide nurse shortage reached 5.9 million in 2018 [1]. At the end of 2022, the number of practicing (assistant) physicians in China was 4.435 million [2], the number of registered nurses was 5.224 million [2] and the physician-to-nurse ratio was only 1:1.18. Following the increase in career choices and employment opportunities for women, the number of women engaged in nursing work is expected to decrease in the future, while the demand for nurses is gradually increasing due to the global acceleration of the aging population and the growth of people’s health care needs [3]. It is especially true for Eastern China, where the rapid economic development brought more employment opportunities; however, due to high living standards and appropriate medical and health conditions, the population’s life expectancy has improved, and longevity has become more prominent [4]. Therefore, Eastern China is already facing a serious shortage of nurses, and maintaining the stability of the nursing team is an important issue for hospital administrators.

Turnover intention has been identified as a prerequisite and predictor of departure [5]. Nurses’ intention to leave varies from country to country; e.g., in Korea, 22.2% of nurses expressed their intention to leave [6], and 43% of Lebanese registered nurses expressed their intention to leave within 12 months [7]. In China, 20.2% of psychiatric nurses reported an intention to leave [8], and 47.3% of Wuhan nurses expressed their intention to leave within half a year [9]. In addition, previous studies have identified multiple factors that affect turnover intentions, including age [10], marriage [11], job stress [12], work-family conflict [13], and career development [14]. Many of these studies have examined the relationship between multiple factors and turnover intentions, but there are still limited studies that have categorized and delved into the personal perspectives of nurses to validate the factors influencing their turnover intentions [8, 13].

In this study, a large sample questionnaire survey was carried out to classify and investigate nurses’ individual, family, and work characteristics to analyze the factors influencing turnover intention.

## Methods

### Subjects

A cross-sectional questionnaire survey of intention to leave was administered to nurses in 13 cities in eastern China between October 1, 2017, and November 30, 2017. In addition, in December 2021, we contacted nurses who resigned between December 2017 and November 2021 to understand their satisfaction with their careers and reasons for leaving.

This study was approved by the Ethics Committee of Nanjing Brain Hospital, Affiliated with Nanjing Medical University (Approval No.2017-KY090), and conducted according to the principles of the Declaration of Helsinki. Each participant was emailed explaining the study, confidentiality, investigator contact information, and electronic informed consent. They were also informed that they could withdraw from the study at any time. All data were anonymized once a form was completed.

The inclusion criteria were (1) agreeing to participate in the survey, (2) holding a registered nurse license, and (3) at least 3 months of hospital work experience. The exclusion criteria were (1) on vacation, (2) leave of absence, or (3) chronic physical or mental diseases that could affect their work.

### Sample

There are 13 prefecture-level cities in Jiangsu Province, and the study included all 13 prefecture-level cities. For each prefecture-level city, one tertiary hospital and one secondary hospital were randomly selected; therefore, 26 hospitals were selected in the 13 prefecture-level cities. The selection of the hospitals was made by the lottery method. The private hospitals were not included. The secondary hospitals were selected first by drawing a name from the bag for each prefecture-level city. Then, the process was repeated for the tertiary hospitals. The nurses were selected in each prefecture-level city using a proportional stratified random sampling method. The method of proportional stratification was to determine the number of nurses drawn from each hospital based on the proportion of the number of nurses selected from two hospitals in each prefecture-level city. Therefore, 2920 nurses were selected to participate in this electronic questionnaire survey.

In the survey of the departing personnel, 102 nurses who had left the hospitals in the above 13 cities were randomly invited to participate in an electronic questionnaire.

### Questionnaire

The electronic questionnaires consisted of three parts: demography, nursing job stressor inventory, and turnover intention questionnaire. The demographic section included personal characteristics, family-related factors, and work-related factors. The nursing job stressor inventory was developed by Xiaomei Li in 1999 [15], with Cronbach’s α of 0.98. The scale includes five dimensions and 35 items focusing on the nursing specialty, workload, work environment, patient, management, etc. Each item is scored on a 4-level scale, where 1 indicates no stress, 2 indicates mild stress, 3 indicates moderate stress, and 4 indicates severe stress. The total score ranges from 35 to 140 points, with higher scores indicating more stress. The Cronbach’s α for was 0.941. Chen et al. divided job stress into four grading states: no stress, mild stress, moderate stress, and severe stress, corresponding to total scores of ≤ 35, 36–70, 71–105, and > 105, respectively [16]. In this study, no stress and mild stress (i.e., total score ≤ 70) were classified as low work stress, while moderate and severe stress (i.e., total score > 70) were classified as high work stress. The turnover intention scale was developed by Michaels and Spector [17] and translated and revised by the Taiwanese scholars Dongrong Lee and Jingyuan Lee [18]. Cronbach’s α was 0.77, and content validity was 67.67%. The scale consists of six items, where items #1 and #6 form dimension I, investigating the possibility of withdrawing from the current job; items #2 and #3 form dimension II, investigating the motivation to find another job; items #4 and #5 form dimension III, investigating the possibility of getting an external job. The questionnaire is scored on a 1–4 scale, where 4 points indicate “often”, 3 points indicate “occasionally”, 2 points indicate “rarely”, and 1 point indicates “never”. The total score ranges from 6 to 24; a higher score indicates a stronger willingness to leave. Cronbach’s was 0.837.

A self-administered questionnaire measured occupational satisfaction. The content contained seven items, including management style, occupational environment, fair treatment, personnel division, opportunities for out-of-office learning, opportunities for promotion, and professional accomplishment, which were divided into very satisfied, satisfied, general, dissatisfied, and very dissatisfied. Total satisfaction = (very satisfied cases + satisfied cases)/total cases × 100%. The reasons for leaving were presented as multiple-choice questions, and the possible options were divided into three categories: individual, family, and work.

### Data collection

The investigators contacted the directors of the selected hospital’s nursing department to explain the study’s purpose, after which they were emailed relevant information. Once approved, these investigators collected the phone and email addresses of potential participants from the selected hospitals and screened them for eligibility by phone. Qualified nurses received emails including study explanations, consent forms, and electronic questionnaires. They were given 4 weeks to complete the questionnaire.

Among the nurses who resigned, the nursing department of each hospital contacted them to present the study. The nursing department provided the phone numbers and email addresses of the willing nurses to participate. Resigned nurses use a lottery method for random sampling, numbering the phone numbers of all willing participants. The research team contacted the nurses, obtained their informed consent, and sent them an email that included research explanations, informed consent, and links to online research questionnaires.

### Exposure variables

Variables related to personal characteristics were sex; age (< 40 years, ≥ 40 years; according to a previous study on willingness to leave [10]); educational level (“Secondary technical certificate”, “Associate degree”, “Bachelor’s degree”, or “Master’s degree”), professional title (“Primary”, “Intermediate”, or “Senior”), position (“Primary nurse”, “General nurse”, “Teaching nurses”, “Assistant management nurse”, “Head nurse”, “Departmental head nurse”, or “Nursing director”), and marital status (“Single”, “Married”, or “Divorced or widowed”).

The family variables were the number of children raised (“0”, “1”, or “≥2”), monthly income (“≤5000 RMB”, “5000-10,000 RMB”, or “≥10,000 RMB”), and the major reason for choosing nursing (“Voluntary”, “Distribution”, or “Parents or family wishes”).

The work-related variables were frequency of night shifts (“Day shift only”, “Night shift ≤ 1/week”, or “Night shift ≥ 2/week”), employment type (“Formal employee” or “Contracted or Third party personnel agency”), number of caregivers (“≤6”, “6–8”, “8–10”, or “≥10”), Part-time job (“No” or “Yes”), and job stress (“total score ≤ 70” or “total score > 70”, according to Chen et al. [16]). Working part-time meant working in another occupation than nursing.

### Outcome variables

We divided turnover into two levels according to the median turnover intention score, i.e., low turnover intention (total score ≤ 15) and high turnover intention (total score > 15).

### Data analysis

The analysis was based on a hierarchical model that describes the relationship between exposure variables and outcomes. First, univariable analyses were performed to explore the relationship between variables and the intention to leave. Next, the statistically significant variables at *P* ≤ 0.05 were selected for binary logistic regression analysis. The stratified selection of independent variables was divided into three determinant blocks: block I (personal characteristics factors), block II (family factors), and block III (work-related factors). Second, we performed multivariable logistic regression according to the stratification method.

Statistical analysis was performed using SPSS 22.0 (IBM, USA). Categorical data were described as numbers and percentages, and percentages between groups were compared using the chi-squared test. Normally distributed measurement data were expressed as means ± standard deviations, and the means were compared between groups using the Z-test. Data with non-normal distribution were presented as medians (interquartile ranges), and the medians were compared between groups using the rank sum test. Univariable analyses were performed using the chi-squared test, and multivariable analysis was performed using a binary logistic stratified regression model, with the effect of each variable expressed as odds ratio (OR) with 95% confidence interval (CI). *P* < 0.05 was considered statistically significant.

## Results

### Nurses’ turnover intention

Among 2796 participants who returned the electronic questionnaires and informed consent forms, 2504 (89.6%) provided valid feedback on all questions. Participants’ intention to leave was scored 15 (12–17), dimension I was scored 5 (3–6), dimension II was scored 5 (4–6), and dimension III was scored 5 (4–6). Among the participants who provided valid feedback on all questions, 1084 (43%) were in the high turnover willingness group.

### Factors influencing nurses’ intention to leave

The results of the univariable analyses showed that five individual-level factors (gender, age, educational level, professional title, and position), three family-level factors (number of children raised, income, and major choice), four work-level factors (frequency of night shifts, employment type, number of caregivers, and part-time job), and job stress affected turnover intention (*P* < 0.05), as shown in Table 1.

**Table 1:** Comparison of characteristics and turnover intention of nurses (*n* = 2504)

Characteristics	Overall%	Turnover intention		*P*
		Low (%)	High (%)	
Gender				
Male	166 (6.6)	80 (5.6)	86 (7.9)	0.022
Female	2338 (93.4)	1340 (94.4)	998 (92.1)	
Age (years)				
≥ 40	323 (12.9)	260 (18.3)	63 (5.8)	< 0.001
< 40	2181 (87.1)	1160 (81.7)	1021 (94.2)	
Educational level				
Master’s degree	2 (0.1)	2 (0.1)	0 (0.0)	< 0.001
Bachelor’s degree	344 (13.7)	191 (13.5)	153 (14.1)	
Associate degree	1293 (51.6)	684 (48.2)	609 (56.2)	
Secondary technical certificate	865 (34.5)	543 (38.2)	322 (29.7)	
Professional title				
Primary	1793 (71.6)	923 (65.0)	870 (80.3)	< 0.001
Intermediate	573 (22.9)	383 (27.0)	190 (17.5)	
Senior	138 (5.5)	114 (8.0)	24 (2.2)	
Position				
Primary nurse (nurse including leader group)	1800 (71.9)	936 (65.9)	864 (79.7)	< 0.001
General nurse (responsible for ward material management, etc.)	46 (1.8)	34 (2.4)	12 (1.1)	
Teaching nurses	110 (4.4)	60 (4.2)	50 (4.6)	
Assistant nurse management (such as office nurse, chief professional nurse, etc.)	222 (8.9)	146 (10.3)	76 (7.0)	
Head nurse (assistant including head nurse)	271 (10.8)	199 (14.0)	72 (6.6)	
Departmental head nurse	31 (1.2)	25 (1.8)	6 (0.6)	
Nursing director	24 (1.0)	20 (1.4)	4 (0.4)	
Marriage				
Divorced or widowed	29 (1.2)	20 (1.4)	9 (0.8)	0.226
Married	1675 (66.9)	960 (67.6)	715 (66.0)	
Single	800 (31.9)	440 (31.0)	360 (33.2)	
Children				
0	1006 (40.2)	543 (38.2)	463 (42.7)	< 0.001
1	1301 (52.0)	783 (55.1)	518 (47.8)	
≥ 2	197 (7.9)	94 (6.6)	103 (9.5)	
Monthly Income (RMB/month)				
≥ 10,000 (USD1572.20)	122 (4.9)	94 (6.6)	28 (2.6)	< 0.001
5000-10,000 (USD786.10-1,572.20)	1016 (40.6)	604 (42.5)	412 (38.0)	
≤ 5000 (USD786.10)	1366 (54.6)	722 (50.8)	644 (59.4)	
Major Choice				
Voluntary	1461 (58.3)	894 (63.0)	567 (52.3)	< 0.001
Distribution	211 (8.4)	135 (9.5)	76 (7.0)	
Parents or family wishes	832 (33.2)	391 (27.5)	441 (40.7)	
Shift status				
Day shift only	750 (30.0)	534 (37.6)	216 (19.9)	< 0.001
Night shift less (≤ 1/week)	576 (23.0)	337 (23.7)	239 (22.0)	
More night shift (≥ 2/week)	1178 (47.0)	549 (38.7)	629 (58.0)	
Employment type				
Formal employee (Service length of the permanent)	863 (34.5)	596 (42.0)	267 (24.6)	< 0.001
Contracted or Third-party personnel agency	1641 (65.5)	824 (58.0)	817 (75.4)	
Care number				
≤ 6	450 (18.0)	296 (20.8)	154 (14.2)	< 0.001
6–8	450 (18.0)	264 (18.6)	186 (17.2)	
8–10	468 (18.7)	240 (16.9)	228 (21.0)	
≥ 10	1136 (45.4)	620 (43.7)	516 (47.6)	
Part-time job				
No.	2424 (96.8)	1389 (97.8)	1035 (95.5)	0.001
Yes	80 (3.2)	31 (2.2)	49 (4.5)	
Job stress	74 (65–84)	71 (62–80)	80 (71–89)	< 0.001
Low	956 (38.2)	708 (49.9)	248 (22.9)	< 0.001
High	1548 (61.8)	712 (50.1)	836 (77.1)	

Binary logistic stratified regression analysis was performed using the level of turnover intention as the dependent variable (low turnover intention = 0, high turnover intention = 1), and 13 statistically significant variables in the univariable analyses were included as independent variables. The following variables affected nurses’ intention to leave: at the individual level, age < 40 years (OR = 1.981, 95% CI: 1.298–3.024); in terms of family factors, nurses who were raising two or more children (OR = 1.617, 95% CI: 1.048–2.493), nurses with a monthly income of USD786.10-1572.20 or < USD786.10 (OR = 1.657, 95% CI: 1.006–2.729; OR = 2.027, 95% CI: 1.223–3.359), and those who were assigned or chose a career according to parental wishes (OR = 1.947, 95% CI: 1.338–2.835; OR = 1.686, 95% CI: 1.395–2.037); in terms of work-related factors, ≤ 1 or ≥ 2 night shifts per week (OR = 1.319, 95% CI: 1.005–1.732; OR = 1.568, 95% CI: 1.208–2.036), contractual or third-party personnel agents (OR = 1.620, 95% CI: 1.270–2.067), and full-time nurse with part-time job (OR = 2.071, 95% CI: 1.239–3.461); high job stress (OR = 3.387, 95% CI: 2.793 to 4.108) (Table 2).

**Table 2:** Analysis of logistic regression models

Dependent variable	Independent variable	*P*	OR (95%CI)	*P*	OR (95%CI)	*P*	OR (95%CI)
		Model 1		Model 2		Model 3	
Turnover intention	Job stress						
	NJSI (1)	< 0.001	3.686(3.070 ∼ 4.425)	< 0.001	3.575(2.967 ∼ 4.307)	< 0.001	3.387(2.793 ∼ 4.108)
	Background characteristics						
	Age (1)	< 0.001	2.573(1.746 ∼ 3.792)	< 0.001	2.569(1.702 ∼ 3.877)	0.002	1.981(1.298 ∼ 3.024)
	Family factors						
	Children			0.042		0.023	
	Children (1)			0.692	0.937(0.678 ∼ 1.294)	0.894	1.023(0.737 ∼ 1.418)
	Children (2)			0.102	1.428(0.932 ∼ 2.188)	0.030	1.617(1.048 ∼ 2.493)
	Income group			0.012		0.007	
	Incomegroup (1)			0.048	1.641(1.005 ∼ 2.679)	0.047	1.657(1.006 ∼ 2.729)
	Incomegroup (2)			0.008	1.953(1.191 ∼ 3.204)	0.006	2.027(1.223 ∼ 3.359)
	Major Choice			< 0.001		< 0.001	
	Major Choice (1)			0.002	1.822(1.255 ∼ 2.645)	0.001	1.947(1.338 ∼ 2.835)
	Major Choice (2)			< 0.001	1.682(1.395 ∼ 2.027)	< 0.001	1.686(1.395 ∼ 2.037)
	Job Characteristics						
	ShiftStat					0.003	
	ShiftStat (1)					0.046	1.319(1.005 ∼ 1.732)
	ShiftStat (2)					0.001	1.568(1.208 ∼ 2.036)
	EmployType (1)					< 0.001	1.620(1.270 ∼ 2.067)
	PartTJob (1)					0.005	2.071(1.239 ∼ 3.461)
	-2 Log likelihood	3085.112		3036.803		2996.291	
	Cox & Snell R Square	0.127		0.144		0.158	
	Nagelkerke R Square	0.171		0.193		0.212	
	Chi-square	340.946		389.255		429.768	

### Analysis of general condition, job satisfaction, and reasons for leaving

A total of 102 retired nurses were contacted. They all gave effective feedback on the questions. Among them, 100% were < 40 years old, 72.5% were nurses who involuntary opted for this career, and 98.0% were nurses with ≥ two night shifts per week and contract nurses or those hired by third-party agencies. The total satisfaction rate of retired nurses with the occupational environment, income, and treatment was 84.9%. The investigation of the reasons for leaving showed that 80.4% of the nurses left due to family reasons (including raising two children, separating from their husbands, low family income, etc.), 39.2% left due to work reasons (including high job stress, frequent night shifts, low professional accomplishment, etc.), and 21.6% left due to other personal reasons (Table 3).

**Table 3:** Investigation of Separating Personnel (*n* = 102)

Item	Overall (%)	Item	Overall (%)
Age(years)		Fair treatment	
≥ 40	0(0.0)	Very satisfied	40(39.2)
< 40	102(100.0)	Satisfied	46(45.1)
Marriage		General	16(15.7)
Divorced or widowed	0(0.0)	Not Satisfied	0(0.0)
Married	50(49.0)	Very dissatisfied	0(0.0)
Single	52(51.0)	Personnel division	
Children		Very satisfied	40(39.2)
0	66(64.7)	Satisfied	50(49.0)
1	28(27.5)	General	10(9.8)
≥ 2	8(7.8)	Not Satisfied	2(2.0)
Major Choice		Very dissatisfied	0(0.0)
Voluntary	28(27.5)	Opportunities for out-of-office learning	
Distribution	36(35.3)	Very satisfied	42(41.2)
Parents or family wishes	38(37.3)	Satisfied	50(49.0)
Shift status		General	10(9.8)
Day shift only	2(2.0)	Not Satisfied	0(0.0)
Night shift less(≤ 1/week)	0(0.0)	Very dissatisfied	0(0.0)
More night shift (≥ 2/week)	100(98.0)	Opportunities for promotion	
Employment type		Very satisfied	42(41.2)
Formal employee (Service length of the permanent)	2(2.0)	Satisfied	48(47.1)
Contracted or Third-party personnel agency	100(98.0)	General	12(11.8)
Part-time job		Not Satisfied	0(0.0)
No	82(80.4)	Very dissatisfied	0(0.0)
Yes	20(19.6)	Sense of personal professional accomplishment and value	
Work environment		Very satisfied	38(37.3)
Very satisfied	46(45.1)	Satisfied	42(41.2)
Satisfied	40(39.2)	General	22(21.6)
General	16(15.7)	Not Satisfied	0(0.0)
Not Satisfied	0(0.0)	Very dissatisfied	0(0.0)
Very dissatisfied	0(0.0)	Job stress	
Income		Low	52(51.0)
Very satisfied	36(35.3)	High	50(49.0)
Satisfied	46(45.1)	Key factors for separation (multiple choice)	
General	16(15.7)	Personal factors	22(21.6)
Not Satisfied	4(3.9)	Family factors	82(80.4)
Very dissatisfied	0(0.0)	Work factors	40(39.2)

## Discussion

This study showed that nurses in Eastern China expressed a relatively high willingness to leave nursing. The survey showed that 43% of nurses expressed their willingness to leave, consistent with previous domestic surveys [13, 19]. Eastern China nurses were more willing to leave than nurses in developed countries. Sasso et al. showed that 35.5% of nurses in Italy had the intention to leave [20], while this was the case for < 25% of nurses in Czechia, Slovakia, South Korea, and other countries [6, 21, 22]. In Japan, 16.2% of nurses were found to have the intention to leave [23]. This variation across countries may be related to the early start of foreign scholars’ research on nurses’ intention to leave. For example, the American scholar McClure proposed the “magnetic hospital” concept in 2002 [24] to solve nursing staff loss by improving the nursing work environment.

This study found that age < 40 was more likely to influence the turnover intention among individual-level influencing factors, which aligns with previous studies [10]. Before the age of 40, female nurses go through different life experiences and physiological stages. In particular, hormone changes during pregnancy and lactation may lead to an inability to concentrate on their work for a long time and to adapt to occupational needs immediately. After China’s two-child policy in 2016 [25], the contradiction between nurses’ career development and marriage and childbearing became even more prominent. Previous studies showed that turnover intention increases when career development is impeded [14]. The 102 retired nurses in this study were all < 40 years of age. Therefore, nurse managers may need to combine the role characteristics of nurses aged < 40 years to understand the influencing factors of their career stagnation, give necessary organizational support, and reduce their willingness to leave. According to some studies, male nurses and nurses with a high educational level are very likely to leave [13, 21]. Yet, this difference was not statistically significant in the present study, which is consistent with the findings of Burmeister et al. [7]. In the development of higher nursing education in China, especially the popularization of adult education [26], the educational level may not be a factor affecting the willingness of Chinese nursing staff to leave their jobs.

The number of children raised is rarely mentioned as a relevant factor in the intention to leave in non-Chinese surveys [7, 23], which may be related to the influence of traditional Chinese ideology and culture. After China liberalized the fertility policy, more and more female nurses were expected to opt to have two or even three children. It would require them to spend more time and energy on their children’s clothing, food, education, etc. [27]., and their focus on life would also shift from work to family, making them less effective in responding to work-related difficulties. Once a family-work conflict occurs, it would increase their willingness to leave [28]. It has been shown that providing parenting support can alleviate the stressors induced by family and work women are commonly facing [29]. Therefore, nursing administrators should arrange the work content according to the different nurses’ specific requirements. In addition, hospitals can provide child trusteeship classes to facilitate nurses to better invest in their work. With the increasing number of two-child and three-child families in China, more nurses may need to be tracked for analysis.

This study showed that nurses with a monthly income of < USD786.10 accounted for 54.6%, and their willingness to leave was 2.027 times higher than nurses with an income of > USD1572.20. Low-income nurses face greater financial constraints [30]. Although some child welfare systems to help raise children have been established in China, compared with various support strategies abroad, the coverage of domestic policies is relatively narrow, and the economic burden of many low-income nurse families is still heavy. Together with rising living costs, such as housing and health care, these women may become anxious and reluctant to patiently wait for the economic growth brought to them by nursing career development. Accordingly, the income level of nurses should be promptly increased through multiple measures to reduce their economic burden.

This study found that personal career choices also impact nurses’ intention to leave. For nurses assigned to their posts or who chose careers following their parents’ wishes, the current profession neither showed their expertise nor aroused their interest. The decline in career satisfaction results in a willingness to leave [31]. Among the nurses who left, 72.5% of the retired nurses reported involuntarily choosing nursing careers in this study. Therefore, managers should cultivate professional interest among nursing staff as much as possible, and more importantly, colleges and universities should provide career guidance for nursing students as early as possible to help them discover and understand their interests and expertise, thus gaining a correct idea of career selection [32].

Previous studies reported that frequent night shifts cause disturbance in nurses’ routines, not only interfering with daily life [33] but also causing health problems, such as fatigue, sleep disorders, and depression [6]. In this study, 98% of the retired nurses had ≥ two night shifts per week. Therefore, nursing administrators should promptly establish a more reasonable and realistic shift management system by formulating reasonable night shift frequency and work content according to seniority.

The survey also revealed that 65.5% of contract or third-party personnel agents nurses were highly willing to leave, consistent with previous studies [34]. Compared with formal nurses, contract or third-party personnel agency nurses have the same work content but a lower salary, causing an increased willingness to leave [35]. Furthermore, 98% of the retired nurses were informal employees. Thus, nursing administrators should gradually narrow the gap between formal and informal nurses in various welfare benefits. Moreover, they should take personalized humanistic care measures so that informal nurses can obtain practical support and help, which in turn might improve their sense of professional belonging.

Part-time employment generally refers to seeking another job outside of one’s position. Compared with professional part-time nurses, there are still a few concerns about full-time nurses engaging in other part-time jobs at home and abroad [36, 37]. The present study found that full-time nurses with part-time jobs were 2.071 times more willing to leave than those without part-time jobs. Among the retired nurses, 19.6% were part-time workers. Once full-time nurses feel that part-time jobs have better development prospects and more income, they quit and concentrate on part-time jobs. Managers should help nurses continuously improve their sense of career accomplishment, as this may help reduce their willingness to leave.

In this study, nurses with high job stress were more willing to leave, similar to previous studies [38] and the conclusions of a systematic review [39]. Increasing nursing workload, frequent nurse-patient disputes, and the gap between professional ideals and reality result in a high-stress environment for nurses [40]. Studies have shown that increased job stress can cause changes in career selection and psychological adjustment of nurses, thus increasing their willingness to leave [21]. Accordingly, hospital administrators should improve nurses’ working environment as much as possible by engaging in a more rational distribution of workload and more methodical setting of work objectives [41], thus reducing their willingness to leave. Of note, the present study was performed in the pre-COVID-19 era, and the pandemic imposes new challenges in nurse retention [42]. The impact of these new challenges on the nurse turnover in Jiangsu will have to be examined.

### Limitations

This study had some limitations. It was a cross-sectional study, and causal relationships could not be established. The research data were all collected through electronic questionnaires, and the accuracy and reliability of the data could not be guaranteed. Additionally, certain information, such as department data, was not collected.

## Conclusion

This survey analyzed the turnover intention and influencing factors in a large sample of nurses in Eastern China, detecting a high willingness to leave. Among the investigated variables, age, the number of children raised, income, personal career choice, frequency of night shift, type of employment, part-time job, and job stress were important factors influencing nurses’ intention to leave. In the future, the willingness of nurses to leave can be reduced by improving the nursing work environment, formulating reasonable career development plans, and improving the treatment level, thus maintaining the stability of the nursing team.

### Electronic supplementary material

Below is the link to the electronic supplementary material.

Supplementary Material 1

Supplementary Material 2

## Data Availability

All data generated or analyzed during this study are included in this article.

## References

[1] Organization WH. State of the world’s nursing 2020: executive summary. 2020.

[2] National Health Commission of the People’s Republic of China DoP. Development and Informatization. Statistical Bulletin on my country’s Health Care Development in 2022. 2023.

[3] Moloney W, Boxall P, Parsons M, Cheung G (2018). Factors predicting registered nurses’ intentions to leave their organization and profession: a job demands-resources framework. J Adv Nurs.

[4] Li Y (2020). Employment countermeasures of China ‘s Road and Population Aging. Int Economic Explor.

[5] Hayes LJ, O’Brien-Pallas L, Duffield C, Shamian J, Buchan J, Hughes F (2012). Nurse turnover: a literature review - an update. Int J Nurs Stud.

[6] Ki J, Ryu J, Baek J, Huh I, Choi-Kwon S. Association between health problems and turnover intention in Shift Work nurses: Health Problem Clustering. Int J Environ Res Public Health. 2020;17. 10.3390/ijerph17124532 10.3390/ijerph17124532 PMC7345885 32599700

[7] Burmeister EA, Kalisch BJ, Xie B, Doumit MAA, Lee E, Ferraresion A (2019). Determinants of nurse absenteeism and intent to leave: an international study. J Nurs Manag.

[8] Jiang F, Zhou H, Rakofsky J, Hu L, Liu T, Wu S (2019). Intention to leave and associated factors among psychiatric nurses in China: a nationwide cross-sectional study. Int J Nurs Stud.

[9] Yang T, Li X, Wang Y, Yao X, Li S (2019). Current status and influencing factors of clinical nurses’ turnover intention in tertiary grade a hospitals in Wuhan. Nurs Manage China.

[10] Labrague LJ, Gloe DS, McEnroe-Petitte DM, Tsaras K, Colet PC (2018). Factors influencing turnover intention among registered nurses in Samar Philippines. Appl Nurs Res.

[11] Lee YW, Dai YT, Park CG, McCreary LL (2013). Predicting quality of work life on nurses’ intention to leave. J Nurs Scholarsh.

[12] Chegini Z, Asghari Jafarabadi M, Kakemam E (2019). Occupational stress, quality of working life and turnover intention amongst nurses. Nurs Crit Care.

[13] Cao J, Jia Z, Zhu C, Li Z, Liu H, Li F (2021). Nurses’ turnover intention and associated factors in general hospitals in China: a cross-sectional study. J Nurs Manag.

[14] Yarbrough S, Martin P, Alfred D, McNeill C (2017). Professional values, job satisfaction, career development, and intent to stay. Nurs Ethics.

[15] mei LX. jun LY. Job stressors and Burnout among Staff nurses. Chin J Nurs. 2000.

[16] Chen M, Xu C, Cheng S. Effect of nursing occupational pressure on the quality of life of contracted nurses. Mod Clin Nurs. 2009.

[17] Michaels CE, Spector PE (1982). Causes of employee turnover: a test of the Mobley, Griffeth, Hand, and Meglino Model. J App Psychol.

[18] Lee G, Lee D, Corner, Conflict (2003). Organizational commitment, and relinquishment willingness under Matrix Organizational structure — — a case study of Industrial Technology Institute Employees. Chin J Manage.

[19] Liu C, Zhang L, Ye W, Zhu J, Cao J, Lu X et al. Job satisfaction and intention to leave: a questionnaire survey of hospital nurses in Shanghai of China. J Clin Nurs. 2012. 10.1111/j.1365-2702.2011.03766.x 21854472

[20] Sasso L, Bagnasco A, Catania G, Zanini M, Aleo G, Watson R (2019). Push and pull factors of nurses’ intention to leave. J Nurs Manag.

[21] de Oliveira DR, Griep RH, Portela LF, Rotenberg L (2017). Intention to leave profession, psychosocial environment and self-rated health among registered nurses from large hospitals in Brazil: a cross-sectional study. BMC Health Serv Res.

[22] Jarosova D, Gurkova E, Palese A, Godeas G, Ziakova K, Song MS (2016). Job satisfaction and leaving intentions of midwives: analysis of a multinational cross-sectional survey. J Nurs Manag.

[23] Saijo Y, Yoshioka E, Kawanishi Y, Nakagi Y, Itoh T, Yoshida T (2016). Relationships of job demand, job control, and social support on intention to leave and depressive symptoms in Japanese nurses. Ind Health.

[24] McClure ML, Hinshaw AS. Magnet hospitals revisited: attraction and retention of professional nurses. Amer Nurses Assn. 2002.

[25] China NHAF (2016). Population and Family Planning Law of the people ‘s Republic of China Chinese. J Practical Rural Doctors.

[26] Wong FKY (2018). Development of advanced nursing practice in China: Act local and think global. Int J Nurs Sci.

[27] Zhang H, Tu J (2020). The working experiences of male nurses in China: implications for male nurse recruitment and retention. J Nurs Manag.

[28] Labrague LJ, Ballad CA, Fronda DC (2021). Predictors and outcomes of work-family conflict among nurses. Int Nurs Rev.

[29] Albanesi S, Olivetti C (2016). Gender roles and medical progress. J Polit Econ.

[30] Chen M, Zhang M, Shi Z (2021). Research Progress of Fertility Support Theory and Practice abroad. Popul J.

[31] Peng P, Yang WF, Liu Y, Chen S, Wang Y, Yang Q (2022). High prevalence and risk factors of dropout intention among Chinese medical postgraduates. Med Educ Online.

[32] Deng W, Feng Z, Yao X, Yang T, Jiang J, Wang B (2021). Occupational identity, job satisfaction and their effects on turnover intention among Chinese paediatricians: a cross-sectional study. BMC Health Serv Res.

[33] Farquharson B, Allan J, Johnston D, Johnston M, Choudhary C, Jones M (2012). Stress amongst nurses working in a healthcare telephone-advice service: relationship with job satisfaction, intention to leave, sickness absence, and performance. J Adv Nurs.

[34] Chen H, Li G, Li M, Lyu L, Zhang T (2018). A cross-sectional study on nurse turnover intention and influencing factors in Jiangsu Province, China. Int J Nurs Sci.

[35] Mosadeghrad AM (2013). Occupational stress and turnover intention: implications for nursing management. Int J Health Policy Manage.

[36] Feng Z, Hongying PI, Qiu S, Meng F, Wei WU, Gao J et al. The setting and management practice in part-time nursing job position. J Nurs Admin. 2019.

[37] Komagata M, Takemura Y, Ichikawa N, Takehara K, Kunie K (2020). Quality of work among part-time nurses and its relationship to job satisfaction and work values: a cross-sectional study. Nurs Health Sci.

[38] Lee EK, Kim JS (2020). Nursing stress factors affecting turnover intention among hospital nurses. Int J Nurs Pract.

[39] Bae SH (2023). Comprehensive assessment of factors contributing to the actual turnover of newly licensed registered nurses working in acute care hospitals: a systematic review. BMC Nurs.

[40] Applebaum D, Fowler S, Fiedler N, Osinubi O, Robson M (2010). The impact of environmental factors on nursing stress, job satisfaction, and turnover intention. J Nurs Admin.

[41] Backman A, Sjögren K, Lövheim H, Edvardsson D (2018). Job strain in nursing homes-exploring the impact of leadership. J Clin Nurs.

[42] Hooper V (2023). Nursing Post Pandemic: the path Forward. J Perianesth Nurs.

