# Supplementary material for: A cross-sectional study on turnover intention of nurses in eastern China
Source: BMC Health Serv Res. 2024 Apr 3;24:425. doi: 10.1186/s12913-024-10849-9 (PMC10993491; doi:10.1186/s12913-024-10849-9)
Supplement: Supplementary file 1 — Supplementary Material 1 [file 12913_2024_10849_MOESM1_ESM.docx]

Supplementary file 1

General information questionnaire

Thank you very much for cooperating with our investigation. The questions are in the form of single selection (please type "√" on the items you think appropriate). This questionnaire is anonymous. The information you provide is only for academic research and is strictly confidential. Sincerely thank you for your support and cooperation!

1、Your gender:

○A、Male ○B、Female

2、Your age:

○A、≥40 ○B、<40

1. Your educational level:

○A、Master’s degree

○B、Bachelor’s degree

○C、Associate degree

○D、Secondary technical certificate

4、Your professional title:

○A、Primary

○B、Intermediate

○C、Senior

5、Your position:

○A、Primary nurse (nurse including leader group)

○B、General nurse (responsible for ward material management, etc.)

○C、Teaching nurses

○D、Assistant nurse management (such as office nurse, chief professional nurse, etc.)

○E、Head nurse (assistant including head nurse)

○F、Departmental head nurse

○G、Nursing director

1. Your marital status:

○A、Divorced or widowed

○B、Married

○C、Single

7、Number of children raised:

○A、0 ○B、1 ○C、≥2

8、Your monthly income (RMB/month):

○A、≥10,000 (USD1572.20)

○B、5000-10,000 (USD786.10-1,572.20)

○C、≤5000 (USD786.10)

9、Your major choice:

○A、Voluntary

○B、Distribution

○C、Parents or family wishes

10、Number of night shift:

○A、Day shift only

○B、Night shift less (≤1/week)

○C、More night shift (≥2/week)

11、Your employment type:

○A、Formal employee (Service length of the permanent)

○B、Contracted or Third-party personnel agency

1. Number of patients to take care of:

○A、≤6

○B、6-8

○C、8-10

○D、≥10

1. Do you have a part-time job:

○A、No ○B、Yes

Thank you again for participating in this survey activity.
