# Supplementary material for: A cross-sectional study on turnover intention of nurses in eastern China
Source: BMC Health Serv Res. 2024 Apr 3;24:425. doi: 10.1186/s12913-024-10849-9 (PMC10993491; doi:10.1186/s12913-024-10849-9)
Supplement: Supplementary file 2 — Supplementary Material 2 [file 12913_2024_10849_MOESM2_ESM.docx]

Supplementary file 2

Occupational satisfaction questionnaire

Thank you very much for cooperating with our investigation. This questionnaire consists of two parts, please type "√" on the items you think appropriate. This questionnaire is anonymous, and any information will be kept strictly confidential. Sincerely thank you for your support and cooperation!

1. Your basic situation

1、Your age:

○A、≥40

○B、<40

1. Your marital status：

○A、Divorced or widowed

○B、Married

○C、Single

1. Number of children raised:

○A、0

○B、1

○C、≥2

4、Your major choice:

○A、Voluntary

○B、Distribution

○C、Parents or family wishes

5、Number of night shift:

○A、Day shift only

○B、Night shift less (≤1/week)

○C、More night shift (≥2/week)

6、Your employment type:

○A、Formal employee (Service length of the permanent)

○B、Contracted or Third-party personnel agency

1. Do you have a part-time job:

○A、No

○B、Yes

1. Your satisfaction with your job
2. Are you satisfied with the management style of the hospital?

○A、Very satisfied

○B、Satisfied

○C、General

○D、Not Satisfied

○E、Very dissatisfied

1. Are you satisfied with the working environment of the hospital?

○A、Very satisfied

○B、Satisfied

○C、General

○D、Not Satisfied

○E、Very dissatisfied

1. Are you satisfied with the income of the hospital?

○A、Very satisfied

○B、Satisfied

○C、General

○D、Not Satisfied

○E、Very dissatisfied

1. Do you think the hospital staff is being treated fairly?

○A、Very satisfied

○B、Satisfied

○C、General

○D、Not Satisfied

○E、Very dissatisfied

1. Are you satisfied with the personnel division of the hospital?

○A、Very satisfied

○B、Satisfied

○C、General

○D、Not Satisfied

○E、Very dissatisfied

1. Are you satisfied with the opportunities for out-of-office learning of the hospital?

○A、Very satisfied

○B、Satisfied

○C、General

○D、Not Satisfied

○E、Very dissatisfied

1. Are you satisfied with the opportunities for promotion?

○A、Very satisfied

○B、Satisfied

○C、General

○D、Not Satisfied

○E、Very dissatisfied

1. Are you satisfied with the sense of personal professional accomplishment and value?

○A、Very satisfied

○B、Satisfied

○C、General

○D、Not Satisfied

○E、Very dissatisfied

9、Key factors for separation (multiple choice)

○A、Personal factors

○B、Family factors

○C、Work factors

Thank you again for participating in this survey activity.
